# Growth enhancement of rice (*Oryza sativa*) by phosphate solubilizing *Gluconacetobacter* sp. (MTCC 8368) and *Burkholderia* sp. (MTCC 8369) under greenhouse conditions

**DOI:** 10.1007/s13205-015-0286-5

**Published:** 2015-03-05

**Authors:** Joseph Stephen, S. Shabanamol, K. S. Rishad, M. S. Jisha

**Affiliations:** Division of Microbiology, School of Biosciences, Mahatma Gandhi University, Kottayam, 686560 Kerala India

**Keywords:** Available phosphorus, Dehydrogenase activity, Grain yield, Phosphatase activity, PGPR

## Abstract

Two indigenous rhizospheric phosphate solubilizing isolates PSB 12 identified as *Gluconacetobacter* sp. (MTCC 8368) and PSB 73 identified as *Burkholderia* sp. (MTCC 8369) were examined for their growth enhancement potential of rice (Jyothi PTB 39) under pot culture assays. The results showed significant impact on microbial count and PSB population, phosphatase and dehydrogenase activity, available phosphorous in the soil, plant nutrient uptake and yield parameters. *Gluconacetobacter* sp. + RP_60_ treatment (30.96 µg PNP g^−1^ soil) retained highest phosphatase activity whereas *Gluconacetobacter* sp. + *Burkholderia* sp. + RP_60_ treatment recorded maximum dehydrogenase activity (38.88 µg TPF g^−1^ soil) after 60 days of treatment. The treatments *Burkholderia* sp. + RP_60_ and *Gluconacetobacter* sp. + RP_60_ produced comparable amount of P and these treatments were statistically at par throughout the growth period. Highest nutrient uptake and yield was noted in *Gluconacetobacter* sp. + *Burkholderia* sp. + RP_60_ treatment. A positive synergistic interaction between strains of *Gluconacetobacter* sp. and *Burkholderia* sp. has been noticed for their plant growth promotion activity. These strains could be of potential to develop as biofertilizers after testing their performance under field conditions either alone or as components of integrated nutrient management systems.

## Introduction

Phosphorus (P) is the second limiting macronutrient for enhanced plant growth and yield next to nitrogen (N). It is involved in the supply, transfer and storage of energy for all biochemical processes inside the plant (Khan et al. 2009). A large pool of inorganic and organic forms of P comprises major P reserve in agricultural soils. In spite of its large demand for increased crop production, only 0.1 % of total soil P exists in a soluble form for plant uptake. This occurs due to the fixation and low solubility of P in soil (Pereira and Castro 2014).

Modern agriculture research is committed for sustainable nutrient management. Different ecological constraints in terms of energy, costs and calamities like emission of poisonous gas, accumulation of heavy metals are associated with industrial methods developed for P deficiency management (Sharma et al. 2013). Microbial mediated P management gains practical attention regarding the ecofriendly and cheapest way of soil P nutrition as they are the key factors in biogeochemical cycles. Phosphate solubilising microorganisms (PSM) are able to carry out solubilization and mineralization of inorganic and organic soil P, respectively, into the bioavailable form for plant root uptake (Rodriguez and Fraga 1999). H_2_PO_4_^−^ and HPO_4_^2−^ are the important forms of P meant for plant assimilation. Major aspect of P cycling and nutrient management is to increase the amount of such free inorganic ions and is largely depending on soil pH. PSM facilitates P solubilization by organic acid production, extracellular enzyme production, chelation and exchange reactions (Khan et al. 2009; Sharma et al. 2013).

The role of rhizosphere microbes as direct plant growth promoters is well established. PSM includes a diverse group of microorganisms including bacteria, fungi and actinomycetes with plant growth promoting abilities like, biological nitrogen fixation, phytohormones production, biocontrol activities etc. other than phosphate solubilization. Bacteria hold foremost position as PSM than fungi and actinomycetes with a population of 1–50 % among total soil microbial populations (Alam et al. 2002). Many phosphate solubilizing bacteria (PSB) belonging to *Pseudomonas*, *Bacillus*, *Rhizobium*, *Burkholderia*, *Serratia, Enterobacter*, *Rhodococcus* and *Arthrobacter* genera have been isolated from soil (Mamta et al. 2010; Karpagam and Nagalakshmi 2014; Pereira and Castro 2014). The competitiveness of a P solubilizing microorganism in natural environments will depend upon its ability to survive and multiply in soil. However, understanding of this part of the use of PSMs is the most limiting factor and it is difficult to predict the behavior and efficacy of the inoculated PSM in a particular location (Gyaneshwar et al. 2002). Such inconsistent performance in diverse conditions reduces interest in experimental observations using microbes. Hence sustainable P nutrient management demands researches on development of potential rhizosphere candidates as PSM.

Rice holds special importance as a global staple food. According to the IRRI knowledge bank, human consumption accounts for 85 % of total rice production worldwide. But the importance of P nutrition to rice plants not yet received considerable attention, compared to N nutrition (Islam et al. 2008). A rise in P nutrient uptake to the plants and the concomitant increase in the growth and yield of rice plants in pot experiments and under field condition have been reported (Son et al. 2007; Panhwar et al. 2011a, b; Vahed et al. 2012). Therefore, systematic scientific investigation can contribute efficient candidates for P nutrient management in rice cultivation. The aim of this study was to evaluate the performance of two PSB *Gluconacetobacter* sp. (MTCC 8368) and *Burkholderia* sp. (MTCC 8369) for the growth, yield and nutrient uptake of rice (*Oryza sativa*) individually and in combination with rock phosphate under green house conditions.

## Materials and methods

### Micro organisms, their origin and culture

The two phosphate solubilizing bacteria PSB 12 *Gluconacetobacter* sp. (MTCC 8368) and PSB 73 *Burkholderia* sp. (MTCC 8369) isolated from the rhizosphere soils of agricultural fields were used in the present study. The identification of the isolates were done on the basis of phenotypic features, whole cell fatty acid methyl ester (FAME) profiles and 16S rDNA typing (Linu et al. 2009). The 16S rDNA sequences of the isolates were deposited in NCBI GenBank under the accession numbers GQ 246872 (PSB 12) and GQ 246871 (PSB 73). A standard phosphate solubilizing bacteria, *Pseudomonas striata* obtained from Indian Agricultural Research Institute, New Delhi was used as reference stain. The cultures were maintained on Pikovskaya’s agar slants at 6 °C in a refrigerator with regular subculturing (Pikovaskaya 1948). For inocula preparation, the cultures were grown separately in Pikovskaya’s broth at 28 ± 2 °C. To obtain bacterial cultures in mid log phase, flasks were incubated for 24 h up to a cell density of 8 × 10^9^ CFU ml^−1^ on a rotary shaker at 30 °C. Bacterial cells were harvested by centrifugation (7000 rpm for 20 min). After removal of the culture medium, the bacterial pellet was washed in sterile water and centrifuged again (7000 rpm for 20 min). Bacterial cells were then resuspended in sterile saline solution and cell density was adjusted to get approximately 8 × 10^9^ CFU ml^−1^ (Van et al. 2000).

### Experimental design and green house treatments

The two efficient phosphate solubilising bacteria from the in vitro experiments were analysed for studying the efficacy on plant growth promotion in vivo under green house condition using pot culture experiments. The experiment was arranged in a complete randomized design (CRD) with three replications per treatment.

### Pot preparation

Soil from rice farming fields (loamy texture, pH 5.0; organic carbon, 1.3 %, available nitrogen, 148 kg ha^−1^; available P 10.2 kg ha^−1^; Dehydrogenase activity: 0.78 µg TPF g^−1^ soil and Phosphatase activity 25 µg PNP g^−1^ soil) was air dried, passed through 2 mm sieve. Fertilizer recommendation include; urea at the rate of 90 kg N ha^−1^, Mussoorie rock phosphate at the rate of 60 kg P_2_O_5_ ha^−1^ (RP_60_) or single superphosphate at the rate of 40 kg P_2_O_5_ ha^−1^ (SP_40_), and muriate of potash at the rate of 11 kg K_2_O ha^−1^. Fertilizers at the rate mentioned above were weighed separately for each pot and mixed with the soil and filled in earthen pots (30 × 30 cm) at the rate of 10 kg per pot. N was applied in three equal splits, as basal dose, at tillering and final dose during panicle initiation. The entire dose of P and K fertilizers was applied as basal dose.

### Soil inoculation and transplantation

The rice seeds (Variety: Jyothi (PTB 39) for the study were collected from Rice Research Station, Kerala Agriculture University, Moncombu, Alleppey, Kerala. Rice seeds were dipped in pre sterilized water in a Petri dish for 18–20 h. Drained off the water and kept the seeds in a closed Petri dish in warm conditions for 2 days. Pre germinated seeds were allowed to grow in nursery bed for 18–20 days by keeping optimum water regime under proper climatic conditions in a green house.

Four, five centimeter dig was made in each pot and each furrow received 5 ml of respective bacterial inoculum. Mixed culture inoculum was prepared by mixing equal quantities of each culture just before application. Roots of 20 days old seedlings were washed several times with sterilized water and transplanted into the dig. The plants were watered twice a day to maintain optimum soil moisture regime and kept under greenhouse condition with ambient irradiance, temperature and air humidity. The crop was harvested after 90 days after transplantation.

The total microbial count and phosphate solubilizing bacterial count were made at 30, 60 and 90 days of crop growth to study the establishment and survival of these bacteria in crop rhizosphere. The populations were enumerated by serial dilution method by plating in nutrient agar and Pikovskaya’s agar media. The available P content of soil samples collected at the same periods were determined by Bray’s II method (Bray and Kurtz 1945). The soil samples were also examined for its Dehydrogenase and Phosphatase activity (Tabatabai and Bremner 1969; Casida 1977).

The plant samples collected at 30, 60 days and the grain and straw collected after harvest were analyzed for total nitrogen and phosphorus by the methods described by Jackson (1973) and N and P uptake were calculated. When the plants were completely mature, harvesting was done. Dry weight of straw and seeds were recorded by drying the material in an oven at 60 °C to a constant weight. In addition, number of panicle per plant, average grain weight per panicle, and number of seeds per panicle were also noted in each case.

The data were subjected to statistical analysis by ‘*F*’ test and the critical difference was calculated by student’s ‘t’ test at 0.05 P level of significance and the means were separated using Duncan’s Multiple Range Test (DMRT).

## Results and discussion

### Effect of PSB on total microbial count and P solubilizing microorganisms in rice rhizosphere

Influence of PSB on crop yield and soil fertility always remains a promising part in the field of sustainable agriculture. Major part of the global cycling of insoluble organic and inorganic soil phosphate is done by microbial P solubilization mechanisms. But it is found that the long term effects of industrial P fertilizers are shown to alter the quality and quantity of microbial activity in soil adversely (Gyaneshwar et al. 2002). The heterotrophic microbial count as well as PSB count in our pot experiments, receiving both the inoculums and RP showed significant increase in microbial count when compared to uninoculated pot soils with RP (Table 1). At 60 DAT highest activity was recorded in *Gluconacetobacter* sp. + *Burkholderia* sp. + RP_60_ treatment (151.33 × 10^5^ CFU ml^−1^). The treatments receiving rock phosphate possessed significantly higher population than non rock phosphate treatment soils. The highest count of PSB (81.67 × 10^5 ^CFU ml^−1^) was detected in *Gluconacetobacter* sp. + *Burkholderia* sp. + RP_60_ treatment followed by 74.33 × 10^5^ CFU ml^−1^ in *Burkholderia* sp. + RP_60_ treatment. Successful adaptation and proliferation of the introduced PSB isolates in natural rhizosphere soil habitats is thus well established. The survival and synergistic effect of inoculated PSB on rhizosphere population of P solubilizers has been reported (Rudresh et al. 2005; Jeong et al. 2013). The rhizosphere PSB isolates PSB 12 and PSB 73 were also studied for their enhanced P solubilization and successful establishment in the soil of pea plants (Linu et al. 2009) and results of this study therefore adds more advantage for using these isolates as soil inoculants.

**Table 1:** Changes in total microbial count, population of PSB and available P in rice rhizosphere as influenced by PSB inoculation

Treatments	Total microbial count on 60 days treatment (×10^5 ^CFU ml^−1^)	Population of PSB on 60 days treatment (×10^5^ CFU ml^−1^)	Available phosphorus	
			60 days	Harvest
Control	24.67^j^	6.33^h^	11.30^g^	10.28^h^
SP_40_	85.67^e^	50.67^d^	44.59^a^	43.90^a^
RP_60_	76.33^f^	47.00^d^	32.64^d^	30.28^d^
*P. striata*	51.67^h^	29.67^g^	25.27^f^	24.16^f,g^
*Gluconacetobacter* sp.	44.33^i^	26.67^g^	24.27^f^	22.99^g^
*Burkholderia* sp.	60.67^g^	37.00^f^	25.29^f^	24.93^f^
*Gluconacetobacter* sp. + *Burkholderia* sp.	64.67^g^	40.33^f^	27.41^e^	26.39^e^
*P. striata* + RP_60_	95.67^d^	61.00^c^	36.05^c^	34.29^c^
*Gluconacetobacter* sp. + RP_60_	112.67^c^	70.00^b,c^	38.05^b^	35.84^b^
*Burkholderia* sp. + RP_60_	133.00^b^	74.33^b^	39.35^b^	36.97^b^
*Gluconacetobacter* sp. + *Burkholderia* sp. + RP_60_	151.33^a^	81.67^a^	45.14^a^	43.13^a^
CD (5 %)	4.49	3.55	0.93	0.97

The mean values with a common letter in the superscript within each column does not differ significantly at 5 % level of significance

Soil P is an indicator of the amount of available P for plant uptake. A significant difference in available P content of soil between treatments was noticed at all stages of plant growth (Table 1) and the effect was more prominent in soil supplemented with RP and PSB. PSB application has been reported to show an increase in the amount of available P in the rhizosphere soil in the findings of Taalab and Badr (2007). At 60 DAT the highest available P content was recorded in *Gluconacetobacter* sp. + *Burkholderia* sp. + RP_60_ treatment (45.14 kg^−1 ^ha) which was statistically at par with that of SP_40_ treatment. The individual treatments of *Burkholderia* sp. and *Gluconacetobacter* sp. with RP_60_ produced comparable amount of P throughout the growth period. The result proves the impact of phosphate mobilization for plant uptake.

The presence or absence of available soil P is directly linked to pH of the soil. A basic mechanism in phosphate solubilization includes production of inorganic and organic acid by PSB. PSB dissolve the soil P through the synthesis of gluconic acid and ketogluconic acid (Nahas 1996). Acid production will lower the rhizosphere pH and it sets for efficient P solubilization. Because, at low pH, H_2_PO_4_^−^, the major soluble form of inorganic P exists in higher amounts (Goldstein 1994). Synthesis of gluconic acids produces hydroxyl and carboxyl groups. These OH and COOH– groups will function as chelating cations Fe^2+^, Al^2+^ and Ca^2+^ associated with insoluble P again leads to lowering of pH. Production of gluconic acid by the introduced isolates used in this study has been reported in our previous studies (Stephen and Jisha 2011).

### Effect of PSB on soil enzyme activity

Present study utilized two phosphatise enzymes, acid phosphatise and dehydrogenase as biological markers to find out the activities of inoculated bacteria in pot soil. While, gluconic acid production shows the solubilization of inorganic phosphates, mineralization of organic phosphates is done by enzymes, especially acid or alkaline phosphatases released by PSM (Sharma et al. 2013). All the inoculated treatments in this study showed substantially high phosphatase activity irrespective of the fact that it was supplemented with RP or not (Table 2). At harvest *Gluconacetobacter* sp. + RP_60_ treatment (30.96 µg PNP g^−1^ soil) retained highest phosphatase activity followed by *Gluconacetobacter* sp. + *Burkholderia* sp. + RP_60_ (29.11 µg PNP g^−1^ soil**)** and *Gluconacetobacter* sp. (27.65 µg PNP g^−1^ soil). Reports can be correlated with Kaur and Reddy (2014) on their studies using PSB for maize crop yield.

**Table 2:** Changes in phosphatase activity and dehydrogenase activity in rice rhizosphere as influenced by PSB inoculation

Treatments	Phosphatase activity (µg PNP g^−1^ soil)		Dehydrogenase activity (µg TPF g^−1^ soil)	
	60 days	Harvest	60 days	Harvest
Control	18.00^k^	16.64^f^	2.39^j^	2.36^i^
SP_40_	25.58^c,d^	23.65^d,e^	16.22^e^	16.15^e^
RP_60_	23.04^d^	22.49^e^	14.93^f^	13.64^f^
*P. striata*	27.14^b,c^	24.18^c,d,e^	10.92^h^	10.24^g^
*Gluconacetobacter* sp.	30.89^a,b^	27.65^b,c^	9.79^i^	8.93^h^
*Burkholderia* sp.	27.96^b,c^	24.72^c,d,e^	11.37^h^	10.60^g^
*Gluconacetobacter* sp. + *Burkholderia* sp.	28.23^b,c^	26.35^c,d^	13.18^g^	12.83^f^
*P. striata* + RP_60_	28.24^b,c^	26.38^b,c,d^	33.06^d^	34.23^d^
*Gluconacetobacter* sp. + RP_60_	34.21^a^	30.96^a^	34.75^c^	35.33^c^
*Burkholderia* sp. + RP_60_	28.79^b,c^	26.46^c,d^	36.28^b^	38.19^b^
*Gluconacetobacter* sp. + *Burkholderia* sp. + RP_60_	31.37^a,b^	29.11^a,b^	38.88^a^	40.20^a^
CD (5 %)	2.12	1.68	0.69	0.66

The mean values with a common letter in the superscript within each column does not differ significantly at 5 % level of significance

Oxidoreductases, such as dehydrogenases, are involved in oxidative process in soils and their activity mainly depends on the metabolic state of soil biota; thus acting as good indicators of the soil microbial activity. Rhizosphere soil from the treatments involving inoculation with *Gluconacetobacter* sp., *Burkholderia* sp. and *Pseudomonas striata* showed significantly higher dehydrogenase activity than the control soil (Table 2). The activity was maximum in *Gluconacetobacter sp*. + *Burkholderia* sp. + RP_60_ (38.88 µg TPF g^−1^ soil**)** and *Burkholderia* sp. + RP_60_ (36.28 µg TPF g^−1^ soil**)**. The effect was more prominent in mixed culture inoculums compared to single culture inoculums revealing the potential positive interaction between *Gluconacetobacter* sp. and *Burkholderia* sp. The measurement of hydrolases provide an early indication of changes in soil fertility since they are related to the mineralization of important nutrient elements required for both plant and microbial growth (Kohler et al. 2007).

### Effect of PSB on nutrient uptake of rice plants

The data pertaining to the effect of phosphate solubilizing bacteria on nutrient uptake of rice crop are presented in Table 3. The inoculation with PSB positively increased the phosphorus content and uptake of plants. These observations strongly confirmed the high P solubilization capacity of the isolates which might have released P from the RP and native inorganic phosphorus due to the action of organic acids and enzymes. P uptake was maximum in *Gluconacetobacter* sp. + *Burkholderia* sp. + RP_60_ treatment (7.970 mg^−1^ plant) after 60 DAT and a substantial increase (15.47 mg^−1^ plant) was observed after 90 DAT. Gupta et al. (2014) reported the enhanced biomass and stevil glycoside production in *Stevia rebaudiana* when treated with PSB and Mussoorie rock phosphate. Comparatively similar response was obtained with other RP supplemented inoculated treatments though the P uptake varied depending on the efficiency of isolates. At harvest period *Burkholderia* sp. + RP_60_ inoculated treatment showed 13.595 mg of P uptake on the other hand its corresponding treatment without RP showed only 6.965 mg of P uptake. Similar observations on the increased P uptake in different crops due to inoculation with P solubilizers have been made by several workers (Jisha and Alagawadi 1996; Taalab and Badr 2007; Sandeep et al. 2008; Panhwar et al. 2012; Kaur and Reddy 2014).

**Table 3:** Nutrient uptake (mg/plant) by rice crop as influenced by PSB inoculation

Treatments	Phosphorus uptake (mg^−1^ plant)		Nitrogen uptake (mg^−1^ plant)	
	60 days	Harvest	60 days	Harvest
Control	0.584^i^	1.428^k^	91.72^k^	150.68^j^
SP_40_	5.405^d^	9.865^e^	319.34^e^	519.48^e^
RP_60_	4.760^e^	8.470^f^	288.85^f^	428.82^f^
*P. striata*	3.265^g^	5.562^i^	197.04^i^	321.28^h^
*Gluconacetobacter* sp.	2.113^h^	4.433^j^	149.64^j^	276.36^i^
*Burkholderia* sp.	3.318^g^	6.965^h^	216.28^h^	366.40^g^
*Gluconacetobacter* sp. + *Burkholderia* sp.	4.456^f^	7.555^g^	264.40^g^	379.11^g^
*P. striata* + RP_60_	5.715^c^	11.585^d^	339.09^d^	571.59^d^
*Gluconacetobacter* sp. + RP_60_	5.700^c^	12.478^c^	392.15^c^	700.51^c^
*Burkholderia* sp. + RP_60_	7.140^b^	13.595^b^	455.89^b^	800.71^b^
*Gluconacetobacter* sp. + *Burkholderia* sp. + RP_60_	7.970^a^	15.470^a^	506.48^a^	888.42^a^
CD (5 %)	0.23	0.19	7.88	9.75

The mean values with a common letter in the superscript within each column does not differ significantly at 5 % level of significance

Soil inoculation augmented the nitrogen uptake by the plant and the trend of nitrogen uptake was similar to that of P uptake. In the RP inoculated series, significant increase was detected with all the PSBs (Table 4). Highest N uptake (888.42 mg^−1^ plant) was noted in *Gluconacetobacter* sp. + *Burkholderia* sp. + RP_60_ treatment. *Burkholderia* sp. + RP_60_ (800.71 mg^−1^ plant) treatment was also equally well followed by *Gluconacetobacter* sp. + RP_60_ (700.51 mg^−1^ plant) and *P. striata* + RP_60_ (571.51 mg^−1^ plant). Reported the increased nitrogen uptake of rice due to inoculation with phosphate solubilizing bacteria has been reported (Sharma and Prasad 2003; Duarah et al. 2011).

**Table 4:** Effect of PSB inoculation on plant yield parameters of rice

Treatments	Average grain weight/panicle (g^−1 ^panicle)	Number of panicles	Number of seeds/panicle	Average panicle length (cm)	Number of tillers/plant	Dry weight of straw (g^−1 ^plant)	Dry weight of seed (g^−1 ^plant)
Control	0.79^d^	3.00^c^	45.75^d^	15.7^g^	3.00^d^	3.08^k^	3.02^k^
SP_40_	1.03^b,c^	6.50^a,b^	58.00^b,c^	19.23^d^	8.25^a,b^	7.01^e^	5.93^d^
RP_60_	1.00^b,c^	6.25^a,b^	57.5^b,c^	18.38^e^	7.25^a,b,c^	5.57^f^	5.71^e^
*P. striata*	0.88^c,d^	4.75^b,c^	50.25^d^	16.00^g^	5.00^c^	5.08^h^	4.78^h^
*Gluconacetobacter* sp.	0.81^d^	4.75^b,c^	49.00^d^	15.74^g^	5.00^c^	4.42^i^	4.28^i^
*Burkholderia* sp.	0.95^b,c,d^	5.25^a,b,c^	51.25^d^	16.45^g^	5.50^c^	5.25^g^	5.11^g^
*Gluconacetobacter* sp. + *Burkholderia* sp.	0.96^b,c,d^	6.00^a,b^	52.5^d^	17.43^f^	6.50^b,c^	5.26^g^	5.28^f^
*P. striata* + RP_60_	1.06^b,c^	6.50^a,b^	59.25^a,b^	19.78^d^	8.25^a,b^	7.21^d^	6.26^c^
*Gluconacetobacter* sp. + RP_60_	1.11^b^	6.75^a,b^	59.75^a,b^	20.93^c^	8.50^a,b^	7.85^c^	6.34^c^
*Burkholderia* sp. + RP_60_	1.12^b^	7.00^a,b^	60.25^a,b^	21.73^a,b^	8.50^a,b^	8.23^b^	6.59^b^
*Gluconacetobacter* sp. + *Burkholderia* sp. + RP_60_	1.33^a^	8.00^a^	64.75^a^	22.01^a^	9.50^a^	8.74^a^	7.01^a^
CD (5 %)	0.09	1.25	3.34	0.43	1.13	0.073	0.11

The mean value with a common letter in the superscript within each column does not differ significantly at 5 % level of significance

### Effect of PSB on yield parameters of rice plants

Rock phosphate in combination with phosphate solubilizing organisms had a greater impact on all the growth and yield parameters of rice viz., dry matter yield, and average grain weight per panicle, number of panicle, number of seeds/panicle, panicle length and number of tillers/plant (Table 4). At 60 DAT highest dry matter production was recorded in *Gluconacetobacter* sp. + *Burkholderia* sp. + RP_60_ treatment (11.07 g^−1^ plant) followed by *Burkholderia* sp. + RP_60_ (10.66 g^−1^ plant). Dry matter yield of all the RP supplied inoculated series was superior over RP_60_ and SP_40_ treatments. The highest grain dry weight was observed in *Gluconacetobacter* sp. + *Burkholderia* sp. + RP_60_ (8.74 g^−1^ plant). On the contrary, treatment receiving *Gluconacetobacter* sp. + *Burkholderia* sp. inoculation without RP gave 5.26 g of seeds. The results are in agreement with previous studies (Sharma and Prasad 2003; Nico et al. 2012; Duarah et al. 2011; Lavakush et al. 2014). The ability of PSB influencing enhanced growth parameters and plant yield of other crops have been studied. Singh et al. (2014) reported the significant uptake of total P in chickpea plants with increased plant growth promotion in terms of seed number and seed weight. Similar reports are published by Gupta et al. (2012) that use of PSB consortium in *Aloe vera* gave higher P uptake and also greatly influences the aloin-A production due to higher plant biomass. Jisha and Alagawadi (1996) reported the nutrient uptake and yield of sorghum (*Sorghum bicolour* L. Moench) was improved by inoculating with phosphate solubilising bacteria. All these reports from diverse crops highlights the crucial factor that growth promotion directly related to the ability of isolates to release P from insoluble RP sources and the other plant growth promoting substances produced by the organism.

## Conclusion

The high P solubilization activity of the introduced PSBs lead to the higher available P content in soil which in turn resulted in increased nutrient uptake of plants and reflected on the growth and yield of rice crops. The plant growth promotion of PSM have been reported to be a combination of several other factors, such as nitrogen fixation, production of plant growth promoting substances, siderophores, HCN, lytic enzymes, competition, control of plant pathogens and by inducing systemic resistance (Pereira and Castro 2014). The PSBs used in this study were already reported for such plant growth promoting attributes (Stephen and Jisha 2011). All the tested biometric parameters showed paramount performance in mixed inocula compared to individual application. The results proves the superiority of the isolates to the standard PGPR strain *Pseudomonas striata* used in this study thereby prospecting the PSBs *Gluconacetobacter* sp. (MTCC 8368) and *Burkholderia* sp (MTCC 8369) as potential microbial inoculants. The results in general provides ample room to use these organisms as potential biofertilizers not only due to its P solubilisation traits but also due to multiple plant growth promoting attributes associated with the bacteria.
